# Diagnosing the Dry Eye Syndrome in modern society and among patients with glaucoma: a prospective study

**Published:** 2020

**Authors:** Nina Bulat, Valeriu Valeriu Cuşnir, Vitalie Procopciuc, Vitalie Cușnir, Nicon Valeriu Cuşnir

**Affiliations:** * Department of Ophthalmology and Optometry, “Nicolae Testemiţanu” State University of Medicine and Pharmacy, Chişinău, Republic of Moldova; ** ”Novamed” Polyvalent Hospital, Chişinău, Republic of Moldova; *** Clinical Hospital of Ministry of Health, Labour, and Social Protection, Chişinău, Republic of Moldova; **** State University of Medicine and Pharmacy, Chişinău, Republic of Moldova

**Keywords:** dry eye syndrome, glaucoma, DEWS, tear film

## Abstract

The dry eye syndrome (DES) is a disease of the ocular surface, which can become a social issue in our country, given the changes in lifestyle as a consequence of the economic and technological developments of the last decade.

A current problem is the prevalence of DES in patients with glaucoma. The glaucoma treatment, especially the prolonged instillation of preservative-containing medicines, is an important factor in DES morbidity, especially in people aged over 40.

In this paper, we presented the findings of our study, which was aimed at assessing the prevalence of DES in the Republic of Moldova and the effect of this impairment on the quality of life. 353 patients (706 eyes) were investigated using the data provided by the visual acuity (VA) and slit-lamp examinations, Schirmer’s test results, and the Ocular Surface Disease Index (OSDI) score. Our findings showed a high estimate of signs and symptoms of DES (67.4%) and their impact on the quality of life. Simultaneously, we aimed to analyze the issue of DES in patients with glaucoma. To this end, we examined 30 cases with primary open angle (POA) glaucoma. We also presented a clinical case, in which the prospect of associating the two pathologies in a patient was considered.

## Relevance of the Subject

The DEWS (Dry Eye Workshop) report defines DES as a multifactorial disorder of the tear film and ocular surface. It is characterized by symptoms of eye discomfort, visual disturbances, tear film inconsistency and possible ocular surface alterations. Actually, this is a dysfunctional tear syndrome, a quantitative or qualitative deterioration of the tear film, lately described as a disorder of the ocular surface [**1**,**3**].

Against the background of economic and technological developments and life changes (environmental pollution, long hours staring at monitors), DES is nowadays increasingly commonplace [**1**,**4**,**5**]. Symptoms of DES are among the most frequent causes of patients’ referral to the ophthalmologist [**5**,**6**]. This issue can greatly limit the patient’s daily activities. DES can be easily diagnosed, but it also requires increased attention from the clinician, because objective signs are not always matching subjective symptoms. The latter can often be underestimated, even when patients encounter real discomfort affecting their quality of life [**7**,**8**].

The impact of DES on the quality of life can be considerable. It is explained by the sensations of pain and irritation with consequences on the state of ocular and general health, the visual performance coupled with the limitation of daily activities like reading, working on the computer or driving. The need for frequent and prolonged instillation of eye lubricants, with the limitation of social and professional activities along with the high cost of treatment, are some other aftereffects of this major public health issue [**1**,**7**].

DES and glaucoma are two common diseases that can oftentimes be found both in the same subject. Glaucoma treatments can cause numerous alterations on the surface of the eye by disturbing the tear secretion, playing a special role in DES morbidity [**2**]. Reciprocally, these pathological changes influence the patient’s compliance due to the emergence of ocular irritation, and consequently the effectiveness of the glaucoma management, both the pharmaceutical treatment (by problems linked to the tolerance of ocular hypotensive medicines), along with the surgical one (by complications of the filtering surgery determined by conjunctival fibrosis) [**9**]. Decreased compliance is a major problem and one of the causes of evolution of optic neuropathy in glaucoma patients. This explains the necessity of the timely detection of DES in patients with glaucoma, as well as the initiation of appropriate and specific treatment of dry eye [**2**].

## Some Statistical Data

According to studies of the prevalence of DES (Women’s Health Study, Physicians Health Study, etc.), 3.23 million American women and 1.68 million American men (totaling 4.91) aged over 50 are estimated to have dry eyes [**1**]. Studies carried out in Spain found 12%, and a much higher prevalence in Asia (27.5-33.7%), i.e. double than the one reported in the U.S. (7.8-14,6), or Australia (5.5-16.6%). The combined data of these epidemiological studies showed that women suffer from ocular dryness more often than men. A meta-analysis of multiple studies shows a prevalence of DES of 5-30% in people aged over 50 [**1**]. These wide variations could be influenced by the different definitions of DES used in different studies. At present, there are no statistical data about the Republic of Moldova concerning the prevalence of DES.

Glaucoma is a common pathology, whose prevalence expands with age from 1% in people aged 40-49 up to 8% in people aged over 80 [**10**].

Additionally, patients administering treatment for glaucoma or ocular hypertension often show signs of eye surface deterioration [**11**]. According to the data of different authors, the frequency of DES in patients with POAG (primary open angle glaucoma) would vary from 11 to 100%, depending on the groups of examined patients (age, sex, ocular hypotensive medication) as well as different criteria for confirming the diagnosis of DES [**2**]. In a study that covered 9,600 patients, more than 40% of the people subjected to the glaucoma treatment had DES symptoms like pain or discomfort upon the instillation of eye drops, foreign body sensation, sensation of ocular dryness, eye burning, and over 20% of the patients had clinical signs of blepharitis, conjunctival hyperemia, or keratitis [**12**]. Leung et al. analyzed 101 cases with POA glaucoma or ocular hypertension. They detected signs and symptoms of DES in more than 50% of the patients, and advanced ocular surface alteration in 27% of the examined people [**13**]. Another study in Germany, covering 20,000 patients, demonstrated that the incidence of dry eye was much higher when 3-4 glaucoma medications were used [**14**]. This frequent association between glaucoma and ocular surface pathology shows a strong connection between these two ocular diseases and their management.

## Pathogenesis and Risk Factors

The pathogenesis of dry eye can be characterized as a vicious circle: once induced, numerous biological mechanisms are triggered and tend to maintain themselves. Thus, the tear film instability causes a chain reaction which leads to inflammation, which consecutively causes the alteration of the quality of tears [**15**,**16**]. The roots of dry eye are complex because there are multiple factors supporting this syndrome: old age (tear secretion decreases with age); hormonal changes in women (pregnancy, menopause, use of oral contraceptives); nutrition (vitamin A deficiency, nutritional intake with low content of omega 3 or an increased ratio of omega 6 compared to omega 3); various diseases of eyelids, incomplete eyelid closure; reduced frequency of blinking (people working on the computer and various video terminals for long hours); conjunctival disorders; prolonged and inadequate contact lenses wearing; side effects of some medicines: anti-allergic medicines, antidepressants, analgesics, cytostatic, anti-migraine and anti-acne medicines; various autoimmune diseases (rheumatoid arthritis, lupus, scleroderma); Sjögren’s syndrome; dermatological diseases (ocular pemphigoid, ocular rosacea); dry, very warm climate with dust and wind; traumas or nerve disorders (Parkinson’s disease); transplantation of hematopoietic stem cells; diabetes, refractive surgery (LASIK); cataract surgery; androgen deficiency; radiotherapy [**1**,**17**].

One cause of the onset of DES is the long-term use of glaucoma eye drops. Multiple studies of conjunctival biopsies, conjunctival prints, or experimental models confirm the toxicity induced to the eye surface by hypotensive eye drops [**14**]. The exact mechanisms of the inflammatory response and/ or of the direct toxicity of the eye drops should be better specified and most likely vary depending on the type and duration of treatment.

According to data of different authors, the main causes of corneal-conjunctival xerosis in glaucoma patients are toxicity of preservatives in ocular hypotensive medications, the pharmacological effect of beta-adrenoblockers and the damaging of the cornea during manipulations for diagnostic purposes (ophthalmotonometry). Preservatives of ophthalmic eye drops and their undesirable effects on the corneal-conjunctival epithelium are among the most studied factors [**18**]. Preservatives in the composition of ophthalmic eye drops are capable of inhibiting the processes of mitosis and can have cytotoxic effects and even cause cellular apoptosis. Also, the prolonged use of eye drops with preservatives can result in squamous metaplasia of epithelial cells [**19**,**20**]. The most commonly used preservative in hypotensive eye drops is benzalkonium chloride with a concentration from 0.005% to 0.02%. The toxic effect of benzalkonium chloride consists in the disturbance of the barrier function of epithelial cells due to intracellular pathological changes. A concentration of 0.1–0.05% of this preservative immediately causes conjunctive cell lysis, a concentration of 0.01% – cell necrosis within 24 hours, and benzalkonium chloride of 0.005 - 0.0001% inhibits the processes of growth and differentiation of cells leading to their apoptosis within 24-72 hours [**21**,**22**]. If the patient with glaucoma already had DES symptoms, administration of hypotensive eye drops, even with a minimal content of preservatives, leads to enhancing the xerotic changes of the cornea [**23**]. Benzalkonium chloride also disrupts the lipid layer in the tear film composition and consequently increases evaporation of tears as well as FL osmolarity with triggered inflammatory process on the eye surface [**24**].

Another factor that would trigger dry eye symptoms in patients with glaucoma is the use of drops containing beta-adrenoblockers. Their regular use stimulates certain alterations in the corneal epithelial cells based on the properties of beta-adrenoblockers of reducing basal and reflective tear secretion, amounting up to 28-36% of the baseline [**25**]. Numerous papers confirm that using beta-adrenoblockers in ophthalmic eye drops leads to tear hyposecretion, disruption of tear film stability, and finally occurrence of xerotic alterations in the corneal and conjunctival epithelium characteristic to the DES [**25**-**27**].

Many authors consider that ocular hypotensive medications play the main role in the development of DES in patients with glaucoma. According to Bonomi et al., after a week of regular use of Timolol maleate 0.25%, punctate keratitis as well as alterations of tear film were reported in every third patient [**28**]. The severity of xerotic changes of the conjunctival epithelium correlates with the frequency of administration of ocular hypotensive medications. As reported by Rossi et al. (2009), DES was found in 11% of glaucoma patients, who were administered hypotensive eye drops once per day, in 39% of those who were administered the medicines twice per day and in 43% of the patients — three times and more per day [**29**]. Comparable results were reported by J. D. Brandt et al. (1991), and later by С. Baudoin et al. (1994-2013), who detected metaplasia of the ocular surface epithelium and changes of the mucin-secreting conjunctival goblet cells, whose degree of severity was directly proportional to the duration and frequency of instillation of hypotensive eye drops [**30**-**31**].

## Purpose of the Work

The purpose of the work was to study the prevalence of DES in the Republic of Moldova and to assess the impact of this disorder on the patient’s quality of life. We also aimed to evaluate the dry eye symptoms in glaucoma patients and to determine the connection between these two pathologies.

## Materials and Methods

In order to assess the prevalence of DES in the Republic of Moldova, 353 patients were investigated, including 139 men and 214 women aged 19-44 (according to the international classification – young people), the professional aspect, and namely the work related to information technologies was characteristic for this group. 218 persons were examined during summer, and 135 during winter. Each patient was subjected to ophthalmologic examination: visual acuity with and without correction, biomicroscopy, fundoscopic examination. The Schirmer’s test (without topical anesthesia) was carried out to assess tear secretion. For the subjective evaluation of patients, the OSDI (Ocular Surface Disease Index) score, a 12-item questionnaire was used to quickly assess ocular irritation symptoms, their impact on daily activities, and their exacerbation in certain environmental conditions [**33**]. According to the pathological history, none of the patients had rheumatologic, endocrinological, or other chronic diseases. Likewise, during the examination, no signs of eye infection were detected in any of the patients.

In order to evaluate the occurrence of dry eye symptoms in glaucoma patients, 30 patients with POA glaucoma were examined. All of them were administered hypotensive eye drops containing preservatives. 56% of them were women, and 44% were men. The average age of the patients in this group was 67 years old, and 21 people (70%) were aged over 60. The disease duration at the moment of diagnosis was 3.7 years on average. 

## Results and Discussions

In total, dry eye symptoms according to the OSDI were detected in 152 persons (43.1%) of the 353 persons from the first group.

The most frequently reported complaints were transient blurred vision along with increased sensitivity to light. Most persons reported that eye problems cause them discomfort while working on the computer, reading or watching TV. Likewise, the patients reported the influence of the environmental conditions on the ocular symptoms, especially of the wind and strong lighting, or increased ocular discomfort in air-conditioned rooms.

According to the Schirmer’s test and/ or the OSDI score, signs and/ or symptoms of DES with mono- or bilateral damage were identified in 238 patients (67.4%).

The Schirmer’s test results showed decreased tear secretion (< or = 15mm/ 5min) in 280 eyes (39.7%) (**Table 1**).

**Table 1:** Results of the Schirmer’s test in patients with lower tear secretion

Test values	Total, 280 eyes	% of eyes, with values of the Schirmer's test ≤ 15
15-11 mm/ 5 min	76	27,1
10-6 mm/ 5 min	105	37,5
5-3 mm/ 5 min	63	22,5
≤ 2 mm/ 5 min	36	12,9

In many cases, a discrepancy between the intensity of the symptoms and the accompanying clinical signs was detected. Thus, 66 (27.7%) of the 238 patients reported dry eye, but the tear secretion was higher than 15 mm/ 5 min, and according to the Schirmer’s test tear hyposecretion was detected in 86 persons (36.1%) who did not present dry eye symptoms (according to the OSDI score).

Signs and/ or symptoms of DES were detected in 65% of the patients examined in summer time, and in 71% of the patients examined in winter time, whereas tear hyposecretion was reported in 42% and 36%, respectively, suggesting a faster evaporation process during the hot period of the year (**Table 2**).

**Table 2:** Reports of signs and symptoms depending on the examination period (summer/ winter)

Examination period	Summer	Winter
Total patients	218	135
Presence of signs and/ or symptoms of dry eye	142 (65%)	96 (71%)
Dry eye symptoms (low/ average/ severe OSDI)	91 (42%)	61 (45%)
Tear hyposecretion (Schirmer’s test ≤ 15mm/ 5min)	183 eyes (42%)	97 eyes (36%)

The gender-based distribution demonstrates that the number of women with signs or symptoms of DES is higher than the number of men (68.9%: 31.1%), as literature data show (**Table 3**).

**Table 3:** Gender-based distribution of patients with Dry Eye Syndrome, compared to the group of asymptomatic patients

Group of patients	Total, patients	Women	Men
Present Signs/ Symptoms of Dry Eye	238	164 (68.9%)	74 (31.1%)
No Signs/ Symptoms of Dry Eye	115	50 (43.5%)	65 (56.5%)

In the group of glaucoma patients, the most frequently reported symptoms were sensation of eye burning – in 19 of the questioned persons (63.3%), sand in the eyes sensation – in 21 persons (70%), eye redness - in 11 persons (36.7%). Other complaints were hypersecretion of tear - 9 (30%) and eyelid itching - 6 (20%). Also, the questioned patients stated that the symptoms are aggravated by certain conditions such as: severe wind – 21 cases (70%), bright sunlight - 15 cases (50%), polluted air - 10 cases (33.3%), air-conditioned rooms - 9 cases (30%), work on the computer - 8 cases (26.7%), heat emitted by various thermal installations – 3 cases (10%), cigarette smoke - in 2 (6.6%) of the questioned persons.

Regarding DES symptoms in the patients with glaucoma, the results of an epidemiological study carried out by Pisella et al. (2002), which covered 4,107 persons, are relevant. The most frequently encountered DES symptoms reported by the patients were [**32**]: excessive discomfort after drops instillation - in 43% of the examined persons, pressure behind eyelids - 40%; foreign body sensation - 31%; sensation of ocular dryness - 23%; excessive reflectory tearing - 21%; eyelid itching - in 18% of the cases. Nordmann et al. analyzed the quality of life in terms of visual deficiencies in 204 treated glaucoma patients. About 93% of them had at least one side effect, such as burning sensation in 25.4% of the cases, transient blurred vision in 20.8%, or hypertearing in 20.2% of examined persons. These local side effects correlated with a worsened quality of life and a higher risk of losing treatment effectiveness [**33**].

The high rate of dry eye symptoms in subjects with POA glaucoma may be also related to older age, or climacteric syndrome in women. A study of Muratova (2005) found out that in women with perimenopause, DES developed more frequently and with a significantly higher intensity on the background of regular instillation of 0.5% Timolol ophthalmic solution [**34**].

## Clinical Case

Patient X, aged 72, with the diagnosis: OD - primary open-angle glaucoma, III B, OS - primary open-angle glaucoma IV B. OU - immature senile cataract. Visual acuity OD/ OS = 0.05/ p.l. uncertain. Intraocular pressure (IOP) OD/ OS = 30/ 29 mmHg.

Duration of time from disease detection - 10 years. Complaints reported by the patient: decreased visual acuity and dry eye. The clinical signs characteristic to dry eye syndrome were assessed objectively: absent tear meniscus, edematous, in accentuated folds conjunctiva, cornea (in OS - degenerative changes of epithelium, in OD – mild arcus senilis).

The Norn’s test (to assess tear film stability) showed: tear film break up time of 3 seconds in OD and immediate break up in OS. The Schirmer’s test results showed decreased tear secretion in both eyes: OD/ OS = 4/ 6 mm/ 5 min. Concomitant diseases - high blood pressure.

The patient receives treatment with hypotensive eye drops for a long time, and namely: Timolol solution 0.5% - 1 drop x 2 times/ day in both eyes. As reported by the patient, the drops were instilled irregularly due to the eye discomfort caused by them. Although the patient showed signs and symptoms of dry eye, no specific treatment was previously prescribed. Surgery was performed in case of OD -extraction of the cataract by phacoemulsification and implantation of intraocular lens, sinus trabeculectomy.

After the surgical treatment, the visual acuity was improved in case of OD from 0.05 to 0.3 (without correction), and the intraocular pressure decreased, in case of OD, from 30 mmHg to 18 mmHg. Medicinal treatment was indicated at home: in case of OD - Diclofenac solution 0.1%, and in case of OS - further administration of Timolol solution 0.5% - 1 drop x 2 times/ day. Taking into account the patient’s complaints of ocular discomfort, as well as the objective signs of ocular dryness, artificial tears of the Systane range were indicated (available in the pharmacy chains in the Republic of Moldova), 1 drop x 3-5 times/ day in both eyes. At the first visit in the postoperative period VA in case of OD is maintained as upon discharge (OD = 0.3), and IOP is compensated - 18 mmHg. In case of OS, a slight decrease in IOP is determined, from 29 mmHg to 25 mmHg. Complaints of ocular dryness were regular. Although the tear film stability and the Schirmer’s test results remained the same, the patient reported improvement in ocular discomfort, with only regular exacerbations of symptoms.

Thus, DES and glaucoma are two pathologies commonly associated in the same patient. An important task for the ophthalmologist is the rational choice of the ocular hypotensive medication, and namely, with high efficacy, but with minimal risk of induction of ocular dryness. In this regard, the instillation of glaucoma eye drops free of preservatives or with a minimum concentration of preservatives is essential in treating glaucoma patients with DES. Lachrymal substitutes, preferably those without preservatives, are very important in the complex treatment of DES in glaucoma patients. Chronic palpebral pathology should not be ignored in case of intolerance to certain topical glaucoma treatments, palpebral hygiene and lipid containing drops being required, and if these measures are not effective, regular antibiotic treatment with cyclins might be useful. Anti-inflammatory treatment is justified by the persistent, DES associated inflammation. It will be treated with local steroidal anti-inflammatory medicines, or, on some cases, local corticosteroids with low intraocular penetration (rimexolone or fluorometholone) may be useful in reducing the iatrogenic risk in patients with glaucoma. Cyclosporine A is an important weapon in treating rebellious ocular dryness and may be a first choice in treating this pathology [**35**]. According to a recent study, the Diquafosol ophthalmic solution 3% is a very effective alternative for treating iatrogenic DES in glaucoma patients [**36**,**37**].

Laser trabeculoplasty, or selective trabeculoplasty, turned out to be highly effective in lowering IOP, and is an alternative method to reduce the medication factor of the ocular surface [**38**]. Glaucoma filtering surgery, either deep non-perforating sclerectomy or trabeculectomy, is also to be considered when removing the medication factor responsible in many cases for the onset or aggravation of DES. Simultaneously, we have to take into account that the ocular surface pathology may cause the failure of filtering surgery by postoperative conjunctival fibrosis [**9**]. Therefore, these interventions will only be performed only after adequate preoperative preparation (non-steroidal anti-inflammatory medications, discontinuation of instillation of eye drops with preservatives, administration of medications such as carbonic anhydrase orally) and application of antimetabolic preparations (mitomycin C) during surgery [**39**].

It is worth noting that often, dry eye symptoms are underestimated in glaucoma patients, as focus is only put on compensation of IOP. Therefore, DES diagnosis becomes defective, and treatment is not initiated on time. The most dangerous consequence of the combination of these two eye disorders is the decrease of the patient’s compliance with the glaucomatous treatment. Because of the irritation and general ocular discomfort right after the instillation of hypotensive eye drops, patients might refuse to further administer them, leading to the progression of glaucomatous optic neuropathy. This emphasizes the importance of prophylaxis, timely detection and appropriate, specific treatment of DES in patients with glaucoma.

## Conclusions

1. Dry Eye Syndrome is an impairment of the ocular surface with a prevalence of 67.4% among young persons and those of working age, aged up to 44, from the Republic of Moldova. Left untreated, it can significantly affect the person’s quality of life and ability to work.

2. A discrepancy was found between the symptoms reported by the subjects and the clinical signs (27.7% - tear hypersecretion, 36.1% - tear hyposecretion), which makes it difficult to evaluate the severity of the disease and requires additional clinical and biological criteria.

3. Dry eye syndrome is a current issue among glaucoma patients, and very likely, it will remain a complicated problem to be solved in today’s generation in 15-20 years.

4. Future important objectives in approaching the glaucoma patient with DES include the reduction of local toxicity of eye drops, the development of glaucoma medications combined with artificial tears, along with the adjustment of specific treatments for ocular surface alterations.
